# Restoration of functional endometrium in an intrauterine adhesion rat model with endometrial stromal cells transplantation

**DOI:** 10.1186/s13287-024-03788-z

**Published:** 2024-06-21

**Authors:** Zhengli Zhou, Xiaomei Wu, Tingwei Chen, Bo Zhang, Wenxin Li, Min Zhou, Jingxue Zhao, E. Dong, Tianqing Li

**Affiliations:** 1 State Key Laboratory of Primate Biomedical Research, Institute of Primate Translational Medicine, Kunming University of Science and Technology, Kunming, 650500 China; 2 Yunnan Key Laboratory of Primate Biomedical Research, Kunming, 650500 China; 3 The First People’s Hospital of Yunnan Province, The Affiliated Hospital of Kunming University of Science and Technology, Kunming, 650031 China

**Keywords:** EnSCs, Immunoregulation, Intrauterine adhesion, Endometrial injury, Therapeutic effect

## Abstract

**Background:**

Intrauterine adhesion (IUA) as a prevalent gynecological disease is developed from infection or trauma. However, therapeutic strategies to repair damaged endometrium are relatively limited. Emerging studies have shed light on the crucial role of endometrial stromal cells (EnSCs) in the process of uterine endometrial regeneration. EnSCs isolated from the uterine endometrium have similar characteristics to mesenchymal stem cells (MSCs). However, it is still unknown whether EnSCs could be used as donor cells to treat IUA. The aim of this study was to evaluate the potential efficacy of EnSCs in treating rat IUA.

**Methods:**

Human EnSCs were isolated from the endometrial tissue of healthy female donors and subjected to extensive expansion and culture in vitro. Immunofluorescence, flow cytometry, cell proliferation assay, trilineage differentiation experiment, and decidualization assay were used to characterize the biological properties of EnSCs. We evaluated the immunoregulatory potential of EnSCs by analyzing their secreted cytokines and conducting bulk RNA sequencing after IFN-γ treatment. After EnSCs were transplanted into the uterine muscle layer in IUA rats, their therapeutic effects and underlying mechanisms were analyzed using histological analysis, Q-PCR, fertility and pregnancy outcome assay, and transcriptome analysis.

**Results:**

We successfully isolated EnSCs from the endometrium of human donors and largely expanded in vitro. EnSCs exhibited characteristics of mesenchymal stem cells and retained responsiveness to sex hormones. Following IFN-γ stimulation, EnSCs upregulated the anti-inflammatory cytokines and activated immunosuppressive molecules. Xenogeneic transplantation of EnSCs successfully repaired injured endometrium and significantly restored the pregnancy rate in IUA rats. Mechanistically, the therapeutic effects of EnSCs on IUA endometrium functioned through anti-inflammation, anti-fibrosis and the secretion of regeneration factor.

**Conclusions:**

Due to their large expansion ability, immunoregulatory properties, and great potential in treating IUA, EnSCs, as a valuable source of donor cells, could offer a potential treatment avenue for injury-induced IUA.

**Supplementary Information:**

The online version contains supplementary material available at 10.1186/s13287-024-03788-z.

## Background

Intrauterine adhesion (IUA), also known as Asherman’s syndrome, is the most frequent gynecological disease characterized by the partial or complete obliteration of the uterine cavity with fibrous tissue attributed by mechanical injury or infection to the basal layer of the endometrium [1, 2]. Patients with IUA experience recurrent miscarriages, infertility, amenorrhea, abnormal placentation, dysmenorrhea and abnormal uterine bleeding, affecting the reproductive health of women, especially those who are seeking to conceive [3, 4]. With the advancement of clinical diagnostic techniques, the diagnostic rate of IUA is gradually increasing. Currently, hysteroscopic adhesiolysis, postoperative placement intrauterine contraceptive devices, low-dose aspirin, and regular estrogen therapy are used as clinical treatments for IUA [5–8]. Nevertheless, these therapies only bring limited benefits for IUA patients and are unable to obtain satisfactory pregnancy outcomes. Especially in patients with severe IUA, the recurrence rate after treatment can be as high as 62.5% 9, 10. Therefore, there is an urgent need to develop more effective and precise approaches for IUA treatment.

Recently, stem cell therapy is a promising treatment to recover dysfunctional endometrium [11]. Mesenchymal stem cells (MSCs) derived from adipose tissue, bone marrow, and umbilical cord sources have been used to repair severely damaged endometrium in rat, rabbit and monkey models [12–15]. Although MSCs have shown promising therapeutic results in the treatment of IUA disease, MSCs are not of endometrial tissue origin, and the search for donor cells derived from endometrial tissue sources may be the most appropriate cellular therapy approach. The endometrium is a unique mucous membrane in the human body, consisting of epithelial, stromal cells, endothelial cells and immune cells. Each female undergoes around 400–500 menstrual cycles in their lifetime, yet it is extremely rare for the injured endometrium during menstruation to develop uterine scarring. This indicates that the endometrium is a highly regenerative tissue regulated by the estrogen-progesterone axis.

Endometrial stromal cells (EnSCs) have the potential to prevent scar formation and have an immunomodulatory capacity, which is responsible for the endometrium’s scarless healing [16]. Furthermore, EnSCs have strong immune modulatory capabilities [17–19]. These indicate that the EnSCs isolated from endometrial tissue may have a stronger contribution to regeneration of injury endometrium. Interestingly, several studies have proved the effectiveness of menstrual blood-derived stromal cells (MEnSCs) in treating IUA [20, 21]. However, MEnSCs are difficult to obtain from human IUA patients. Thus, it may be an ideal method for repairing IUA by autologous transplantation using EnSCs obtained from IUA patients.

Here, we successfully isolated EnSCs from endometrium of human donor and largely expanded. EnSCs have characteristic of mesenchymal stem cells and retained sex hormones responsiveness. Upon IFN-γ stimulation, EnSCs exhibited an immunoregulatory function. After transplantation, EnSCs successfully repaired injured endometrium and significantly improved the pregnancy rate in IUA rats. Mechanistically, EnSCs grafts induced regeneration mediated by anti-inflammation, anti-fibrosis and regeneration factors secretion.

## Methods

The work has been reported in line with the ARRIVE guidelines 2.0.

### Animals

All rats experiment protocols were conformed with the Guide for the Care and Use of Laboratory Animals and were approved by the ethical committee of the Kunming University of Science and Technology (PZWH (dian) K2022-0009). All animals were purchased from experimental animal center of Kunming Medical University and housed at laboratory animal center of the Kunming University of Science and Technology. All rats were fed under standard living conditions (22 ± 1 °C, 12-hour light/12-hour dark, and free access to water and food pellets). Rats were monitored daily after surgery and then rats with excessive distress, including excessive weight loss, lethargy, and loss of thermoregulation, were euthanized.

### Human tissue collection

This study was approved by the Ethics Committee of First People’s Hospital of Yunnan Province Affiliated to Kunming University of Science and Technology (ethics number: KHLL2021-KY049) and has been performed in accordance with the principles of the Declaration of Helsinki. All donors signed informed consents for voluntary donation of endometrium at the First People’s Hospital of Yunnan Province. A total of 5 Endometrial specimens in the proliferation stage of the menstrual cycle without any abnormalities or malignancies were obtained from women of reproductive age (mean age: 30), who underwent hysteroscopic surgery for benign gynecological diseases, such as hysteromyoma and infertility. Characteristics of each donor are provided in supplementary Table 1. Patients undergoing endometrial malignant or precancerous lesions, endometrial polyps, endometrial hyperplasia, uterine cavity adhesion and hormone therapy were excluded from the sample collection. Endometrial tissue was collected by curettage.

### Endometrium tissue dissociation and EnSCs isolation

The endometrial tissue was dissociated through a two-stage dissociation protocol as previously described [22]. Upon receiving the endometrial samples, which were placed in a 15 ml Falcon tube with DMEM medium and transported in a chilled carrier, the tissues were initially rinsed with saline solution to remove excess mucus, ensuring the overall cleanliness of the tissue. The endometrial tissue was minced into 1 mm [3] pieces on a petri dish using surgical scissors and incubated dish in a tissue digestion solution containing Type IV collagenase, dispase, and DNAse I on a rocking platform (50–70 rpm depending on the model) for 15–20 min at 37 °C to assist with digestion. After brightfield microscopic examination, single cells were observed, while the glandular epithelial fragments still remained, the digestion was terminated. When presenting red blood cell contamination, using ACK Lysis Buffer to remove blood cell. Epithelial cells were separated using a 40 μm cell strainer. Stromal cells located beneath the strainer were then collected. EnSCs isolated from those collected endometrium was subsequently used to cell culture, transcriptome sequencing, and cellular therapy in animal experiments.

### Culture and expansion of EnSCs

For the harvested stromal cells, they were seeded onto regular plastic culture dishes at a density of 1 × 10^4^ cells per square centimeter (cm²) and cultured in primary complete stromal medium (PCSM). PCSM contains DMEM/F-12 + GlutaMAX™ basic medium (GIBCO), supplemented with 10% FBS (GIBCO), 2mM l-glutamine (GIBCO), 100 IU/ml penicillin + 100 mg/ml streptomycin (GIBCO), 5 µg/ml insulin (Sigma-Aldrich), 25 ng/ml hydrocortisone (Sigma-Aldrich), 0.125 ng/ml EGF (GIBCO). The above dishes were placed in an incubator at 37 °C, 95% air, 5%CO_2_. When the cell confluence rate reaches 80-90%, cells were digested with Trypsin-EDTA (Gibco) to serially passaged. After passage to 3–5 passages, the cells were purified and frozen with cell freezing medium in -80℃.

### Immunofluorescence staining of EnSCs and endometrium tissue

EnSCs were grown to a certain density in 96 well plates and fixed in 4% (w/v) paraformaldehyde for 15 min. After washing with PBS and permeabilization with 0.1% Triton X-100 (Sigma-Aldrich) for 10 min at 4 °C and blocking with 5% bovine serum albumin for 30 min at room temperature. Cells were stained with the primary antibody vimentin (1:300, Abcam, ab137321) and cytokeratin 7 (CK7) (1:200, Abcam, ab181598) at 4℃ overnight. After washed for three times, the secondary antibody and DAPI were incubated with cells for 1 h at 37℃ in the dark. For immunofluorescent(IF) staining of rats’ endometrial tissue, samples were firstly fixed in 4% paraformaldehyde for 24 h and then embedded in OCT and cut into 5 μm slices for IF staining. For intracellular target proteins staining, sections were fixed, permeabilized, blocked and then incubated with the primary antibodies E-cadherin (abcam,1:400), vimentin (abcam,1:300) or CD31(ab24590,1:100) overnight at 4 °C. Fluorescein-conjugated secondary antibody were incubated, followed by addition of DAPI to stain the nucleus. The IF results were observed and photographed under a confocal fluorescence microscope (Leica, Sp8) and analyzed with LAS X software.

### Flow cytometric analysis

The adherent P3 generation of EnSCs cells, digested into single cells, were washed twice with pre-chilled PBS. Then, cells were incubated with related cell surface flow cytometry antibodies at 4 °C in dark for 30 min, subsequently washed twice with cold PBS, resuspended in 500µL PBS and analyzed by a flow cytometer (FACSAria II, BD, USA). These markers included human positive MSC maker CD90-FITC, CD105- PerCP-Cy™5.5, CD73-APC, CD44-PE, negative MSC cocktail marker (CD45-PE, CD34-PE, CD11b-PE, CD19-PE and HLA-DR-PE (BD, MSC analysis kit, 562245) and two pairs of classic markers for endometrial stromal stem cells. That is CD146-FITC (BioLegend, 361012), PDGFR-β-APC (BioLegend, 323608) and SUSD2-PE (BioLegend, 327406). Then EnSCs phenotype were characterized by flow cytometry for cell surface markers including CD90, CD105, CD44, CD73 and CD146, PDGFR-β, SUSD2. In all experiments, isotype antibodies were also used as negative controls.

### Cell proliferation assay of EnSCs

Once the EnSCs isolated, the passage 0 cells (P0) were serially passaged to P20 (passage every 3 days). Cell numbers were quantified at each passage. The cell growth curve was drawn according to the absolute cell number of each passage by software Graph Prism 7.

### Adipogenic, osteogenic and chondrogenic differentiation of EnSCs

For adipogenic differentiation, EnSCs were seeded into into a 6-well plate at a density of 200,000 cells/well. Adipogenic Differentiation was induced in an adipogenic culture medium prepared according to the product instructions. After that, lipid droplets in cells were stained with Oil Red-O and visualized in an inverted light microscope. For osteogenic differentiation, seeded cells in a 6-well plate was incubated with Osteogenic differentiation medium. Differentiation was assessed using Alizarin Red staining (osteogenic). For chondrogenic differentiation, cells were cultured in the chondrogenic induction medium and then stained by Alcian blue solution and observe chondrogenic differentiation under a light microscope.

### Decidualization of EnSCs

EnSCs were seeded into 12-well plates at the appropriate density, and hormone induction was initiated when the cells reached 50% confluence. EnSCs were induced for decidualization for 5 days via two distinct pathways: E2 + P4 + cAMP and P4 + cAMP, using 10 nM estradiol (E2, Sigma, E1024), 1 µM MPA (P4,Sigma, PHR1589), and 1 µM cAMP (2’-O-dibutyryladenosine 3’,5’-cyclic monophosphate sodium salt; Sigma, D0627). Each treatment was repeated in three wells. Samples from the decidualization experiments were harvested using Trizol (Gibco,15596026) to extract RNA for downstream Q-PCR analysis.

### EnSCs responsiveness to inflammatory cytokine IFN-γ

P3 EnSCs from three patients, cultured in 6-well plate, with confluence reached to 70%, were exposed to 50 ng/ml human interferon gamma (IFN-γ) (peprotech) for 2 days in complete primary stromal cell media (PSCM). Complete media without IFN-γ served as a control. Cells from the two groups were harvested by 0.05% Trypsin-EDTA digestion (ThermoFisher Scientific) and subsequently used for bulk RNA-seq.

### Establishment of the EnSCs-GFP cell line

The GFP lentivirus was packaged using the conventional three plasmid system method as previous described [23]. 293T cells with 80% confluence were transfected using packaging plasmids (psPAX-2), envelope plasmids (pMD2.G), and GFP lentivirus plasmids (pWPXLd) with the cationic polymer HighGene transfection reagent (ABclonal, RM09014) for lentivirus packaging. Virus supernatants were collected at 48 h and 72 h post-transfection. The GFP lentivirus, filtered through a 0.22 μm membrane, was used to infect EnSCs, establishing the EnSCs-GFP cell line.

### IUA rat model establishment

The IUA rat models were established by comparing three different mechanical curettage methods and one chemical injury method to assess the optimal modeling approach for uterine endometrial injury. 10-week-old rats weighing 250–300 g in the estrus phase were randomly divided into four groups, each corresponding to one of four different modeling methods: knife scraping, needle scraping, knife excision, and ethanol treatment. Briefly, after anesthesia with Isoflflurane(0–5%) using Small Animal Anesthesia Machine(RWD Life Science Co.,Ltd), the abdominal wall and cavity were surgically opened and the bilateral uteri were exposed under sterile conditions. For knife scraping methods, the uteri were cut longitudinally in the middle of the uterus to expose the endometrium, creating a 3.5 cm incision. The endometrium was then scraped with a blade until the surface of the uterine cavity became rough. For needle scraping, a 0.3 cm longitudinal incision was created 1.0 cm above the cervix of the uterus. A No. 18 needle (inner diameter: 1.50 mm; outer diameter: 1.80 mm; needle tube length: 35.5 mm) was inserted into the uterine cavity through the incision and scraped back and forth along the entire layer of the uterine cavity for no fewer than 50 times until the uterine exhibited obvious congestion reaction and roughness. For the alcohol group, the upper and lower ends of the uterus were clamped with vascular clamps. One milliliter of 95% ethanol was slowly injected into the upper end of the uterine cavity, leaving the uterine cavity filled for 15 min.

### Transplantation of EnSCs to treat IUAs in rats

Rats were randomly assigned to 3 groups: the sham-operated group, the IUA group, the EnSCs group (*n* = 4–5 in each group for each experiment). Different treatment measures were performed in each group after the establishment of the IUA model. In the sham-operated group, the abdominal cavity was surgically exposed without any manipulation of the uterus. In the IUA group, no treatment was administered. In the EnSCs group, 300 µL of DMEM basic medium containing 2 × 10^6^ cells were transplanted directly via intrauterine injection into two sites: the uterine muscle layer or uterine cavity.

### H&E and Masson staining

At the appointed time, rats were sacrificed and bilateral uterine were resected. Uterine tissue was fixed in 4% paraformaldehyde with standard paraffin embedding. Paraffin-embedded tissue samples were sectioned into 5-µm slices. The tissue sections were deparaffinized in xylene at room temperature and subsequently rehydrated through a descending ethanol series (100% for 5 min, 95% for 1 min, 80% for 5 min and 75% for 5 min). The HE and Masson staining were performed according to routine procedures. Tissue sections were mounted with neutral resin and observed under optical microscopy (Nikon, NIKON ECLIPSE E100). The number of endometrial glands and the degree of fibrosis were determined based on the results of HE and Masson staining, respectively. Image J (Image in Java, USA) software was used for statistical analysis of the average proportion of each group.

### Immunohistochemical analysis

All slides underwent the same standard procedures as in HE staining before performing antigen retrieval. Then sections were fixed in 3% hydrogen peroxide solution for 15 min to block endogenous peroxidase reactivity and blocked in goat serum for 30 min at 37 °C. Then slides were incubated with the primary antibodies to estrogen receptor (ESR, 1:300, Servicebio, GB13025), anti-progesterone receptor (PGR, 1:300, Servicebio, GB11262), anti-platelet-derived growth factor B (PDGFB, 1:200, Servicebio, GB11261) and anti-insulin-like growth factor-1 (IGF1, 1: 1000, Servicebio, GB11248) overnight at 4℃. Then washed using PBS and incubated with secondary antibody labeled by HRP for 2 h at room temperature followed by PBS wash. Finally, the 3,3-diaminobenzidine tetrahydrochloride (Servicebio, G1211) staining was used to visualize target antigen location. Slides were counterstained with hematoxylin for 2 min. The results were imaged using a microscope (CIC. XSP-C204). Staining intensities were quantified using Image-Pro Plus software (Media Cybernetics).

### Quantitative RT-PCR

Total RNA was extracted from 50 ∼ 100 mg of excised tissue using TRIzol™ reagent (Gibco, 15596026). One microgram of harvested total RNA was then reverse transcribed to generate cDNA following the manufacturer’s protocol (Takara, RR047A). The gene expression levels of TGF-β1, α-SMA, and IGF1 were determined by qPCR using the 2^−ΔΔCq^ method with Power SYBR-Green Mastermix in a 20 µL volume (Applied Biosystems, USA), with the GAPDH gene used as a reference. The primer sequences are listed in supplementary Table 2.

### Fertility test

Since the 28th day of treatment, female rats in each group were housed with healthy, fertile male rats at a ratio of 2:1 to assess the function of the scarred uterine horns. The rats were euthanized 18 days after the presence of vaginal plugs, and each uterine horn was collected to examine the number of implanted embryos. The embryo development of each group was photographed.

### RNA sequencing and bioinformatics analysis

Once the RNA quality extracted from both EnSCs and rat uteri samples meets the required standards, the standard RNA-seq procedure is followed. This includes reverse transcription into cDNA, adapter ligation, amplification, and sequencing to obtain the raw data.

#### Gene expression analysis

The processing and analysis of this collected data were conducted using R software. To map reads and quantify transcript expression levels, the HISAT2 and StringTie-Ballgown workflows were employed. The paired-end clean reads were aligned to the Rattus norvegicus (UCSC rn6) reference genome using StringTie software (v2.1.1). After the alignments, the FPKM values were calculated using Ballgown (v2.22.0).

#### Principal component analysis (PCA)

Genes with FPKM values less than 1 were filtered across all samples. The remaining genes were selected for dimensional reduction by principal component analysis (PCA) and displayed using the R package ggplot2 (v3.4.4).

#### Differential gene analysis

Firstly, before conducting differential analysis, genes with FPKM values less than 1 were filtered across all samples. The remaining genes were subjected to differential expression analysis using the R package Deseq2 (v1.30.1). The DEGs were defined as genes with a p-value < 0.05 and |log2FC| > 1.

#### Weighted correlation network analysis (WGCNA)

Gene co-expression network analysis was specifically performed on rat endometrium tissues using the R package WGCNA (1.72.5). The expression matrix was restricted to differentially expression genes including EnSCs group VS Injury group, as well as injury group vs sham group at different time points. Hierarchical Clustering was used to filter the outlier samples. Then, the optimal soft threshold for adjacency computation was graphically determined, and we plotted module detection via dynamic tree cutting. Genes with highly similar expression profiles were grouped into one module based on the “blockwise Modules” function. The minimum module size is set to 30, and the ‘mergeCutHeight’ is set to 0.30. The soft threshold set to 10. To identify key modules associated with EnSCs transplantation, module eigengene and group were calculated by Pearson correlation and visualized using heat maps of module and groups. Modules with a higher correlation were chosen for further analysis. The top 30 genes with the highest KME value in the module was displayed in fitting curves over the time points to show changes.

#### Enrichment analysis

Genes with kME > 0.65 within each module underwent enrichment analysis using online websites (KOBAS: http://kobas.cbi.pku.edu.cn/).

#### Gene Set Enrichment Analysis (GSEA)

The differential expression gene generated in Injury VS Control and Injury VS EnSCs was subjected to GSEA functional enrichment using R package “clusterProfiler”. The Normalized Enrichment Scores (NES) of the selected pathways was showed by heatmaps using R package " pheatmap (v1.0.12)” to compare difference between the two groups.

### Statistical analysis

One-way analysis of variance (ANOVA) was used to analyze study variables among groups, and significance of difference between two groups was compared by Independent-Samples T Test. Data are shown mean ± SD, and *P < 0.05, **P < 0.01, and ***P < 0.001 were considered to indicate a statistically significant difference. All statistical analyses were performed using GraphPad Prism 9 software (San Diego, CA, USA).

## Results

### Expansion and identification of endometrial stromal cells (EnSCs)

**Fig. 1:**
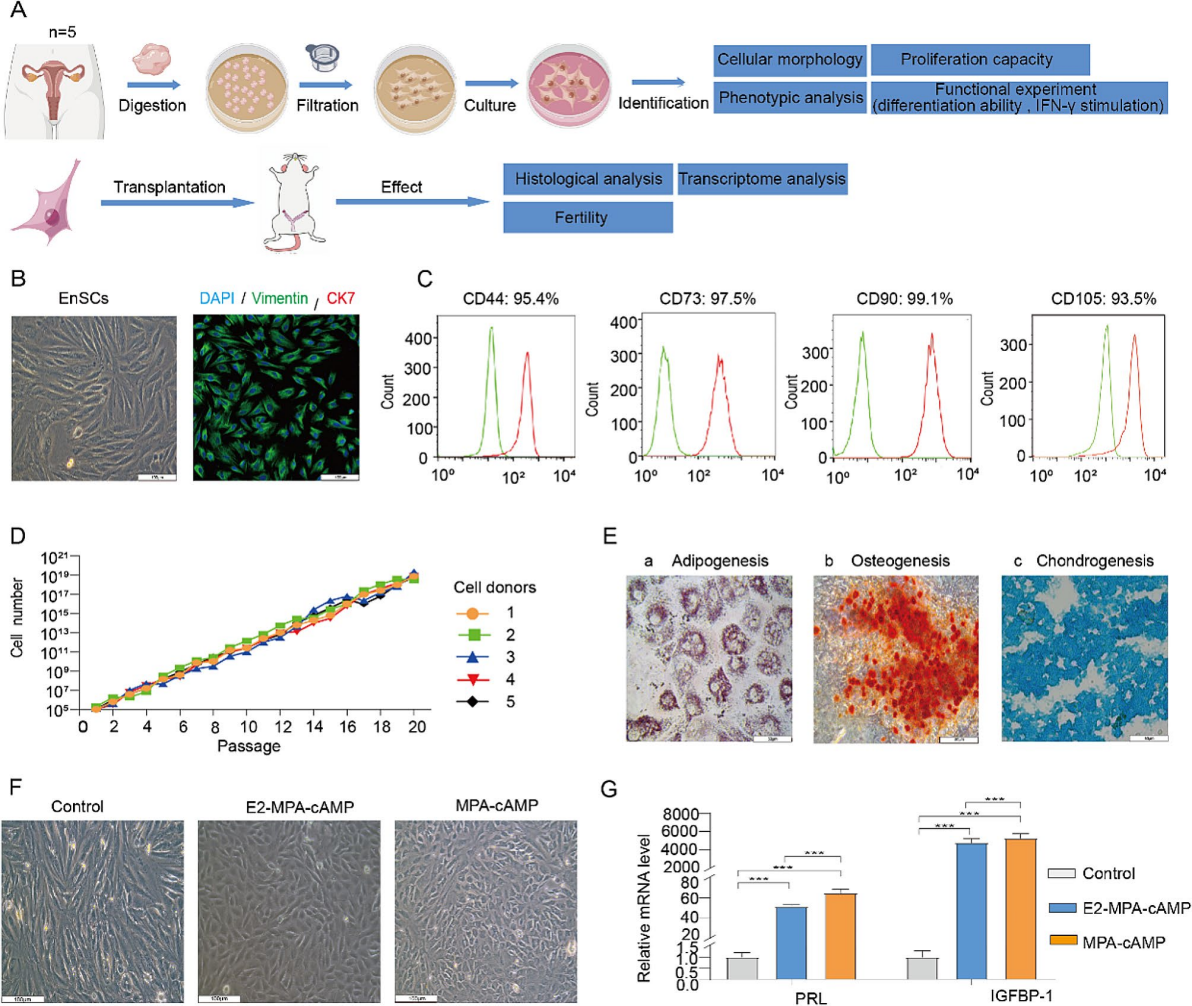
The biological properties of endometrial stromal cells (EnSCs). **A** A schematic diagram to illustrate the isolation, expansion, and identification of the EnSCs (*n* = 5) and their transplantation into the IUA rat model resulting from endometrial injury. **B** The morphology of EnSCs at passage 3 (left panel) and the mesenchymal characteristics of EnSCs stained by immunofluorescence (right panel). Scale bars: 100 μm. **C** Phenotype of EnSCs analyzed by flow cytometry using marker CD44 (95.4%), CD73 (97.5%), CD90 (99.1%), and CD105 (93.5%). **D** Proliferation curves of EnSCs from 5 cell donors from passage 1 to passage 20. **E** Trilineage differentiation assays of EnSCs(P3). Adipogenic (a), osteoblastic (b), and chondrogenic (c) Scale bars: 50 μm. **F** Morphological changes in EnSCs during decidualization under conditions of E2 + MPA + cAMP and MPA + cAMP, respectively. Scale bars: 100 μm. **G** The expression levels of the two decidualization markers, PRL and IGFBP-1, were determined using Q-PCR. Data were represented as mean ± SD (*n* = 5). One-way ANOVA for comparisons of different groups. ***p < 0.001

Primary EnSCs were isolated as described in “Methods” section (Fig. 1A). To optimize the culture condition of EnSCs, we added three additional components, insulin, EGF, and hydrocortisone into the conventional EnSCs medium consisting of the DMEM basal medium supplemented with 10% serum. The isolated EnSCs were cultured in the optimized medium to adequately preserve their endometrial characteristics in vitro, ensuring a more stable and rapid expansion of EnSCs. EnSCs exhibited a typical mesenchymal morphology (Fig. 1B). Immunostaining of stromal marker VIM and epithelial marker CK7 revealed the mesenchymal characteristics of EnSCs (Fig. 1B). After several passages, EnSCs exhibited a homogeneous spindle-shape morphology, and were detected with MSC common markers (CD44, CD105, CD90 and CD73) by flow cytometry. Flow cytometry analysis of EnSCs from 5 donors showed that EnSCs consistently exhibited high expression levels of MSC markers, with a positivity rate exceeding 90% after passage 3 (P3) (Fig. 1C and S1A). To monitor the proliferation ability of EnSCs over long-term cultures, we quantified the number of cell from five different clinical samples across passages 1 to 20, respectively. The cell proliferation curves showed that EnSCs have strong and stable proliferation capacity in long-term cultures (Fig. 1D). Next, we tested the trilineage differentiation potential of EnSCs into adipocytes, osteocytes and chondrocytes using Oil Red O staining, Alizarin Red staining and Alcian Blue staining. The results showed that EnSCs can differentiate into adipocytes, osteocytes and chondrocytes (Fig. 1E). To further validate whether the histological characteristics of EnSCs were maintained during culture, we assessed their decidualization responses to estradiol (E2), progestin (Medroxyprogesterone acetate-MPA, P4) and cAMP in high-passage (beyond 10 passages) EnSCs [24, 25]. Upon hormone treatment, EnSCs underwent a transformation of cell morphology from spindle-shaped to epithelial-like (Fig. 1F). Q-PCR results showed that both E2 + MPA + cAMP and MPA + cAMP treatments significantly upregulated decidualization markers *IGFBP-1* and *PRL* (Fig. 1G). Together, the long-term expanded EnSCs in vitro sustain both the characteristics of the MSCs and endometrial properties, exhibiting a decidualization response to hormone treatment.

### Immunomodulatory potential of EnSCs after IFN-γ stimulation

**Fig. 2:**
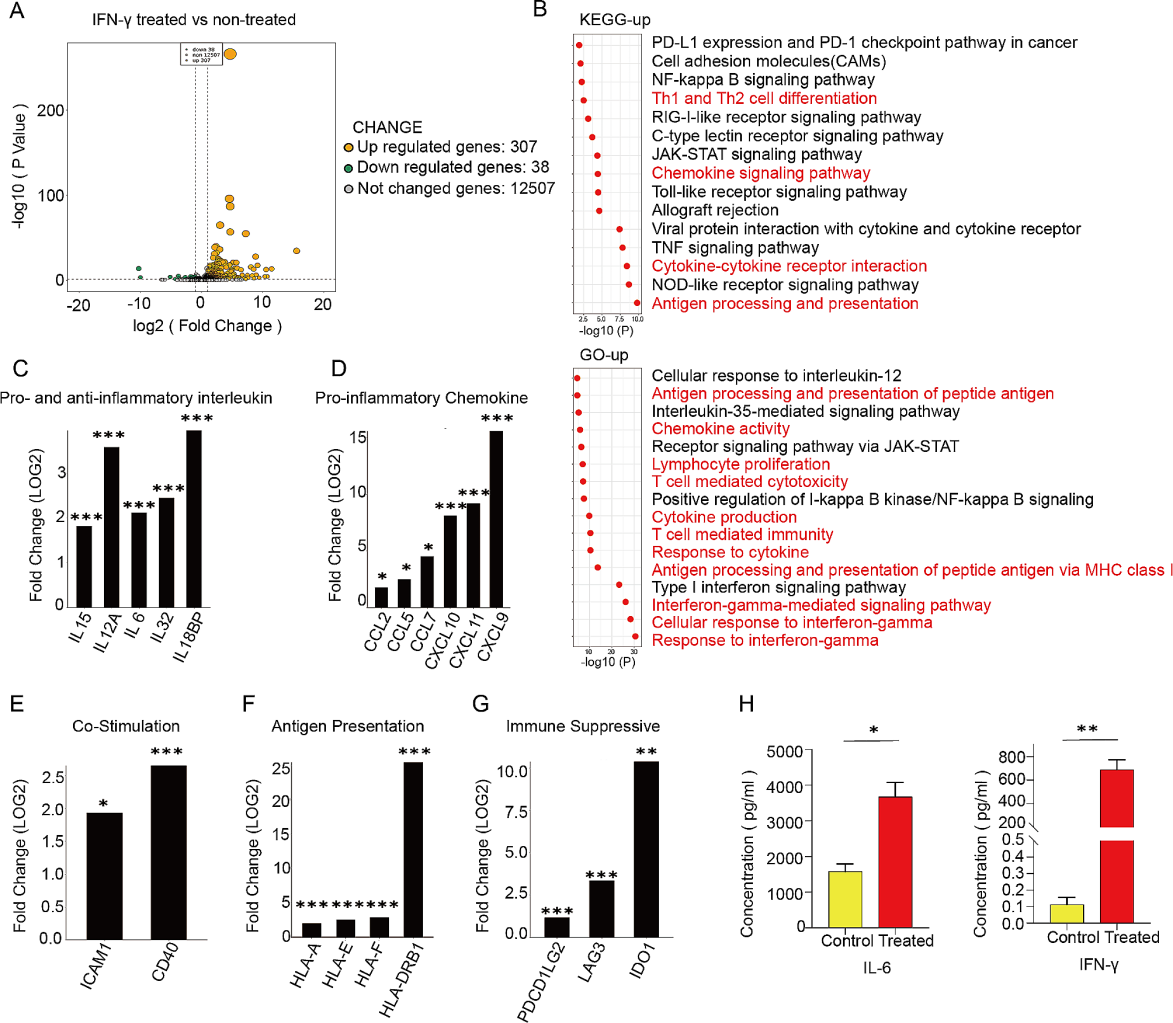
Immunomodulatory properties of EnSCs after IFN-γ stimulation. **A** The volcano plot for the differentially expressed genes between IFN-γ treated EnSCs vs. non-treated group EnSCs (307 up-regulated and 38 down-regulated genes). *n* = 3. **B** Representative KEGG- pathway (upper panel) and GO terms (bottom panel) enriched in IFN-γ treated EnSCs. **C**-**G** The Fold change (LOG2) of some representative genes in EnSCs after IFN-γ treatment, including pro-inflammatory and anti-inflammatory interleukin (**C**), pro-inflammatory chemokine (**D**), antigen presentation-associated co-stimulatory molecules (**E**), antigen presentation (**F**) and immunosuppressive molecules (**G**). *n* = 3. *p < 0.05, **p < 0.01, ***p < 0.001. **H** The secretion concentrations of Th1 cytokines IFN-γ and Th2 cytokines IL-6 in the cultured EnSCs, with or without IFN-γ treatment, were assessed using Cytometric Bead Array (CBA) assays. Data were represented as mean ± SD (*n* = 3). Unpaired Student’s t test. *p < 0.05, **p < 0.01

IFN-γ is one of the established licensing factors for priming MSC immunosuppressive properties [26]. To assess the immunomodulatory properties of EnSCs, we stimulated cultured EnSCs (*n* = 3 donors) with or without the pro-inflammatory cytokine IFN-γ for 2 days, and then performed bulk RNA transcriptome analysis. Volcano plots showed IFN-γ treatment significantly upregulated 307 genes and downregulated 38 genes compared to the non-treatment control (Fig. 2A). The significant upregulation of CXCL9, CXCL10, and CXCL11 in IFN-γ-treated EnSCs indicated that IFN-γ signaling pathway activated in EnSCs (Fig. S1B). Furthermore, KEGG pathway enrichment analysis and GO function annotation of upregulated DEGs revealed that IFN-γ primed EnSCs were mainly enriched in antigen presentation pathways, cytokine-cytokine receptor pathway, chemokine signaling pathway, Th1 and Th2 cell differentiation, PD-L1 and PD-1 checkpoint pathway (Fig. 2B). The results together suggested that IFN-γ primed EnSCs displayed potent immunoregulatory activities by transcriptionally stimulating the expression of various genes involved in immune regulation and other biological activities.

Specifically, the immune response ability of EnSCs was mainly dependent on both paracrine activity and cell-to-cell contact. For paracrine activity, IFN-γ also upregulates anti-inflammatory interleukins such as IL-6, IL-32 and IL18-BP (Fig. 2C). Notably, when exposed to IFN-γ, pro-inflammatory interleukins such as IL-15 and IL-12A were upregulated in EnSCs (Fig. 2C). IFN-γ also promoted the expression of chemokines such as CXCL9, CXCL10, CXCL11, CCL5, CCL2 and CCL7 (Fig. 2D). For cell-to-cell contact molecules, antigen presentation-associated co-stimulatory molecules ICAM1 and CD40 were also significantly elevated (Fig. 2E). Besides, human leukocyte antigen (HLA) class I and II molecules were significantly upregulated in IFN-γ treated EnSCs group (Fig. 2F). PD-L1 and PDL-2, as well as IDO1, were immunosuppressive molecules affecting T cell function. Transcriptomics data showed that IFN-γ treatment significantly up-regulated the expression of immunosuppressive molecules such as IDO, PD-L2 and LAG3 (Fig. 2G).

To further assess the cytokine secretion profile of EnSCs following IFN-γ stimulation, we also employed Cytometric Bead Array (CBA) technology to detect the expression levels of Th1 cytokines IFN-γ and TNF-α, which shift naïve T cell to Th1, as well as Th2 cytokines IL-4, IL-6, and IL-10, which shift naïve T cells to Th2. The results revealed that following IFN-γ treatment, the anti-inflammatory Th2 cytokine IL-6 was markedly upregulated (Fig. 2H). However, we failed to detect any increase in IL-4, IL-10, and TNF-α secretion after IFN-γ treatment (data not shown). These results indicate that EnSCs may have immunoregulatory functions to shift naïve T cell to Th2 in an inflammatory environment upon IFN-γ induction.

### Trans-myometrial injection of EnSCs facilitates the survival of transplanted cells within the uterus

To establish an appropriate IUA model in rats, we used four methods: mechanical damage by blade scraping, needle scratching, blade incision, and chemical injury caused by 95% ethanol. After injury, their corresponding endometrium structural changes were evaluated, especially the high columnar luminal epithelium and degree of fibrosis, by histological analysis including hematoxylin and eosin (H&E) and Masson’s trichrome staining, respectively. We observed that the injury caused by needle scraping method resulted in a uterine cavity structure that more closely resembles the clinical IUA phenotypes, with a narrower and entangled uterine cavity (Fig. S2A, B). Subsequently, we found that the injured endometrium caused by needle scraping displayed uterine adhesion, showcasing a narrower uterine cavity, and notable reductions in endometrial thickness and gland numbers on the 7th day post-needle scratching (Fig. S2C, D). Additionally, increased fibrotic areas within the endometrium were observed (Fig. S2E, F). Therefore, we established the rat IUA model using needle scratching to evaluate the therapeutic effects of EnSCs.

Structurally, the uterus is connected to the external environment, which raises the possibility of transplanted cells exiting the uterus. To explore an effective method of cell transplantation, we compared the cell survival rates after GFP-labeled EnSCs were injected into uterine muscle layer and uterine cavity, respectively (Fig. S2G). GFP signals in the uterus were detected on days 1, 3, 5, 7, and 14 post-transplantations (Fig. S2H). Human nuclear antibody was used to co-stain the GFP signals to confirm these transplanted cells. In rats with uterine cavity injection, GFP^+^ cells were only found in the tissue close to endometrial cavity within 3 days, whereas obvious GFP^+^ cells were observed up to 7 days post injection into the uterine muscle layer (Fig. S2H). The results showed that the uterine muscle layer injection is beneficial for cell survival and localization in endometrium. Therefore, we adopted uterine muscle layer injection for subsequent experiments.

### Transplantation of EnSCs ameliorates endometrial fibrosis and recovers the uterine luminal epithelium in the IUA model rats

**Fig. 3:**
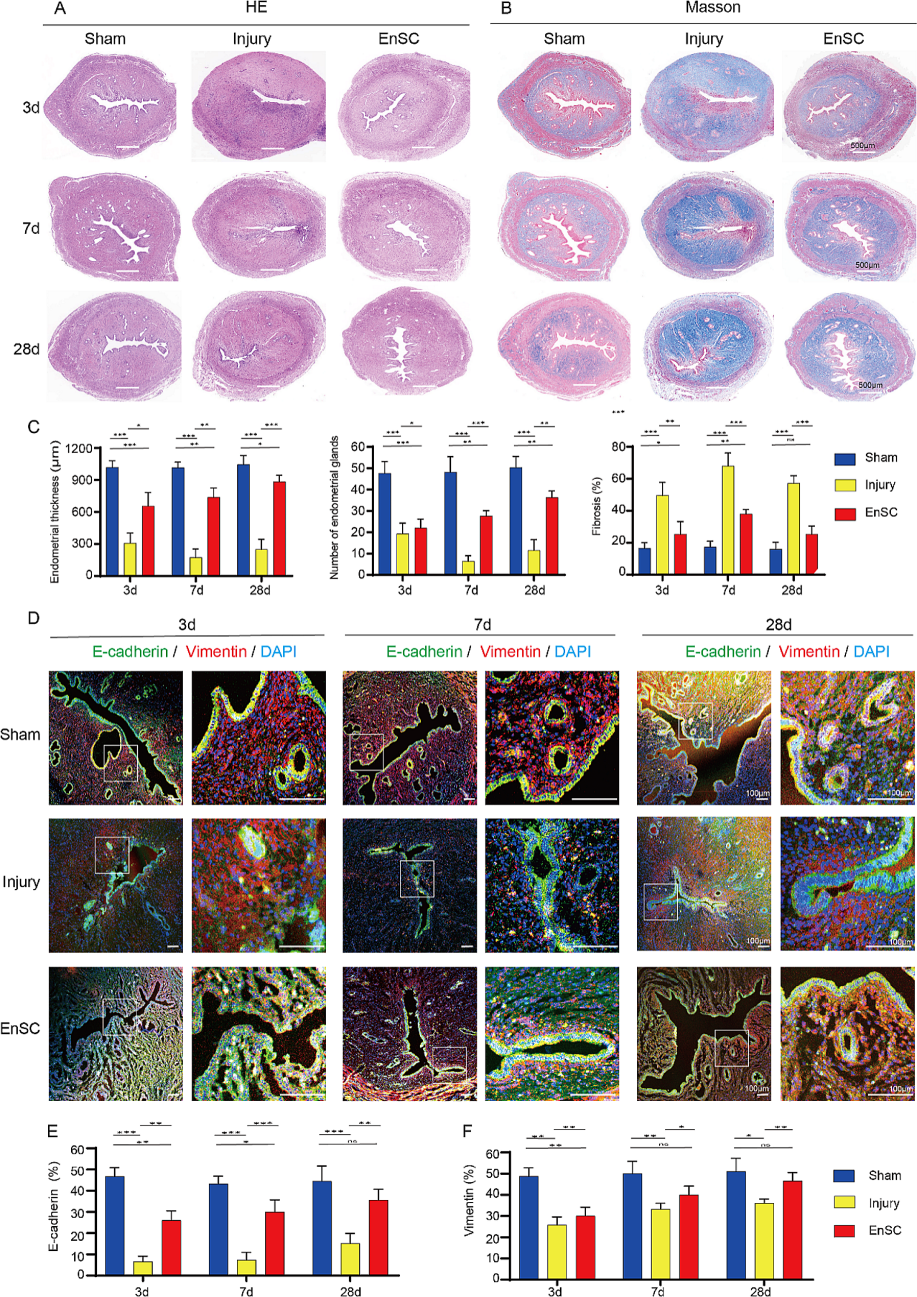
EnSCs transplantation repaired the uterine epithelium in IUA rats. **A**, **B** H&E staining (**A**) and Masson’s trichrome staining (**B**) of uteri in the sham group, injury group and EnSC-treatment group rats on 3-,7- and 28d post-injury, respectively. Scale bars: 500 μm. **C** Quantification of endometrial thickness, endometrial gland numbers and fibrosis degree in rats from the sham group, the injury group and the EnSC treatment group at 3-, 7-, and 28-days post-injury. Data are means ± SD (*n* = 8). One-way ANOVA. *p < 0.05, **p < 0.01, ***p < 0.001. **D** Representative immunostaining images of E-cadherin and Vimentin in uteri from different groups at 3-, 7-, and 28-days post-injury, respectively. Scale bars: 100 μm. **E, F** Quantification of E-cadherin- (E) and Vimentin- (F) positive cells in the sham group, injury group, and EnSC treatment group at 3-,7- and 28d post-injury. Data are represented as means ± SD (*n* = 6). One-way ANOVA. *p < 0.05, **p < 0.01, ***p < 0.001, ns, no significance

Next, we examined whether EnSCs have the capacity to repair the injured endometrium in vivo. To determine the most appropriate dose of EnSCs, we compared the therapeutic effects of 1 × 10^6 cells and 2 × 10^6 cells. The dose of 2 × 10^6 cells yielded greater therapeutic benefits than that of 1 × 10^6 cells (Fig. S3A-E). Therefore, we opted to utilize 2 × 10^6 cells in subsequent experiments. To comprehensively evaluate the restorative effects of EnSCs on the damaged endometrium, we injected EnSCs into the uterine muscle layer tissues, and collected endometrium samples at three different time points post-cell therapy: 3 days, 7 days and 28 days. The results of histological analysis of H&E, Masson’s trichrome staining as well as quantification of endometrial thickness, gland number and fibrosis area showed increases in endometrial thickness and gland number and a decrease in fibrosis in the EnSCs transplantation group, compared to those in the injury group at all three time points (Fig. 3A-C). These findings collectively show that the EnSC grafts restored the injured endometrium morphology and decreased the fibrotic lesion formation.

Since E-cadherin (E-cad) was specifically expressed in the luminal epithelial cells in the uterine cavity and interstitial glandular cells and Vimentin (Vim) positive cells were almost exclusively endometrial stromal cells [27, 28] (Fig. 3D), we therefore annotated E-cad^+^ cells and Vim^+^ cells into epithelial cells and stromal cells, respectively. In the sham group, E-cad^+^ luminal epithelium cells lining the uterine cavity remained structurally continuous and intact (Fig. 3D). In the injury group, the luminal epithelium detached and became discontinuous, resulting in disrupted epithelial polarity and a decline in E-cad^+^ epithelial cell numbers (Fig. 3D, E). In contrast, on 3, 7 and 28 days post-EnSCs transplantation, we observed an integrated luminal epithelium with an increased presence of scattered glands within the stroma and an increase in E-cad^+^ epithelial cell numbers (Fig. 3D, E). Furthermore, on 3, 7 and 28 days, Vim^+^ stromal cells were significantly increased after treatment with EnSCs (Fig. 3D, F). Together, EnSCs transplantation significantly recovered the IUA endometrium to its normal structure, which is composed of regularly arranged columnar luminal epithelium, and enhanced the numbers of glands and stromal cells.

### Transplantation of EnSCs restores the fertility in IUA rats

**Fig. 4:**
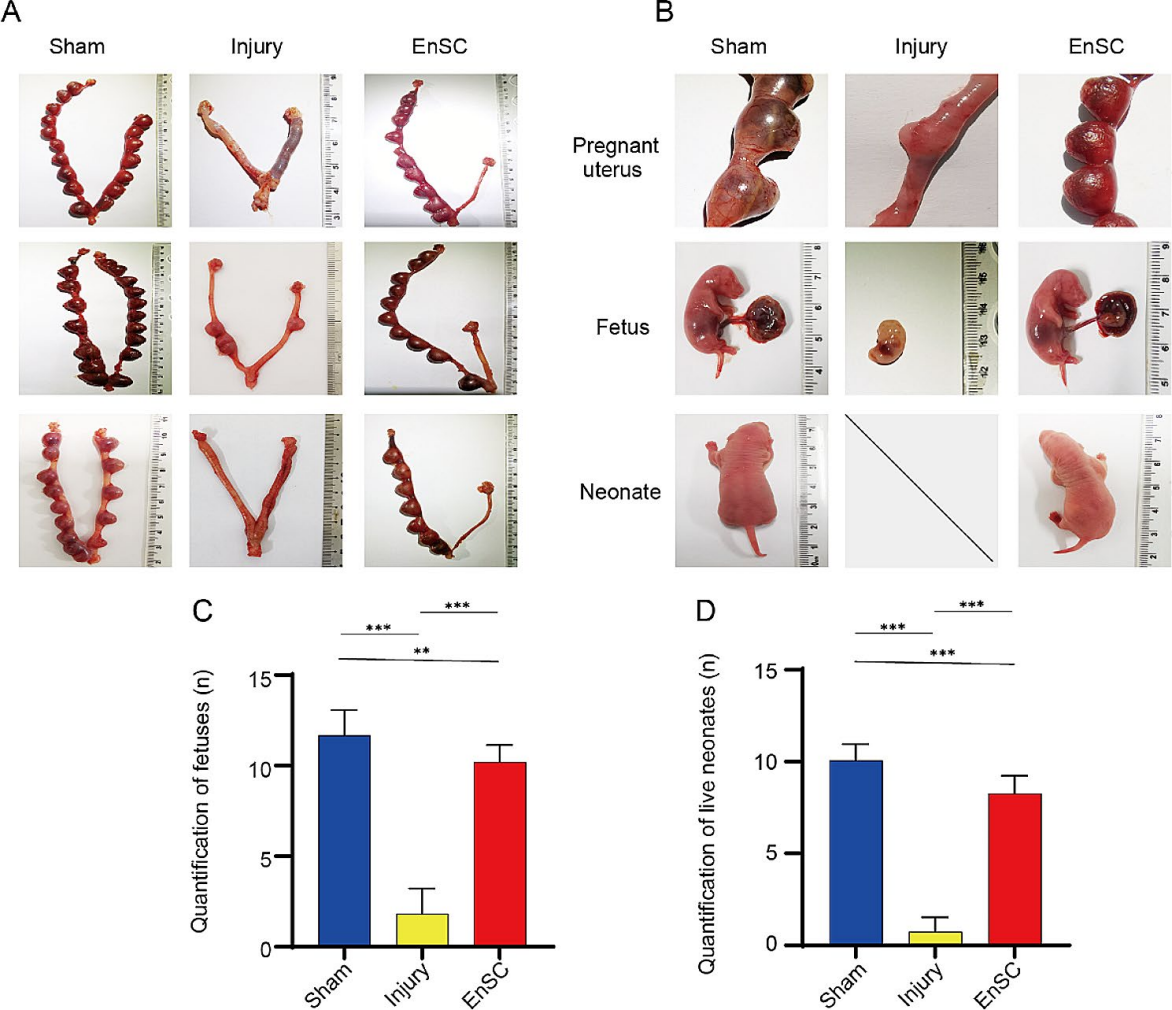
EnSCs transplantation restored the fertility in IUA rats. **A** Representative pregnancy images of rats in the different groups on 30d post-injury. In both the EnSC and injury groups, the endometrium in bilateral uteri were damaged, with only the right uteri receiving EnSCs transplantation in the EnSC group. The rats in the sham group underwent laparotomy but did not undergo any procedures on the bilateral uterus. **B** Representative fetuses and neonates from different group rats. **C**, **D** Quantification of fetuses (**C**) and live neonates (**D**) in the right uteri of rats from different group. Data are represented as mean ± SD (*n* = 10). One-way ANOVA. **p < 0.01, ***p < 0.001

To investigate the functional improvement of injured endometrium after EnSCs transplantation, 30 female rats (*n* = 10 in each group) were bred on 30d after EnSCs transplantation. In the sham-operated group, all uteri exhibited conception, whereas in the injury group, only one rat became pregnant with few embryos present, and other injured uteri lost the ability to conceive (Fig. 4A). In contrast, a significant increase in the pregnancy rate was detected in the EnSCs treatment group, but no conception was observed in the damaged uteri without EnSCs treatment group (Fig. 4A). Besides, we observed a significant difference in the number of fetuses between the cell transplantation and the non-transplantation group (Fig. 4B). Next, we evaluated the number of viable fetuses from pregnant rats and observed an increased count of viable neonates in EnSCs treatment group (Fig. 4C, D). These findings indicated that EnSCs are capable of restoring the injured endometrium and dramatically improving fertility in IUA rats.

### EnSCs grafts recovered endometrium by anti-inflammation, anti-fibrosis and regeneration factor secretion

**Fig. 5:**
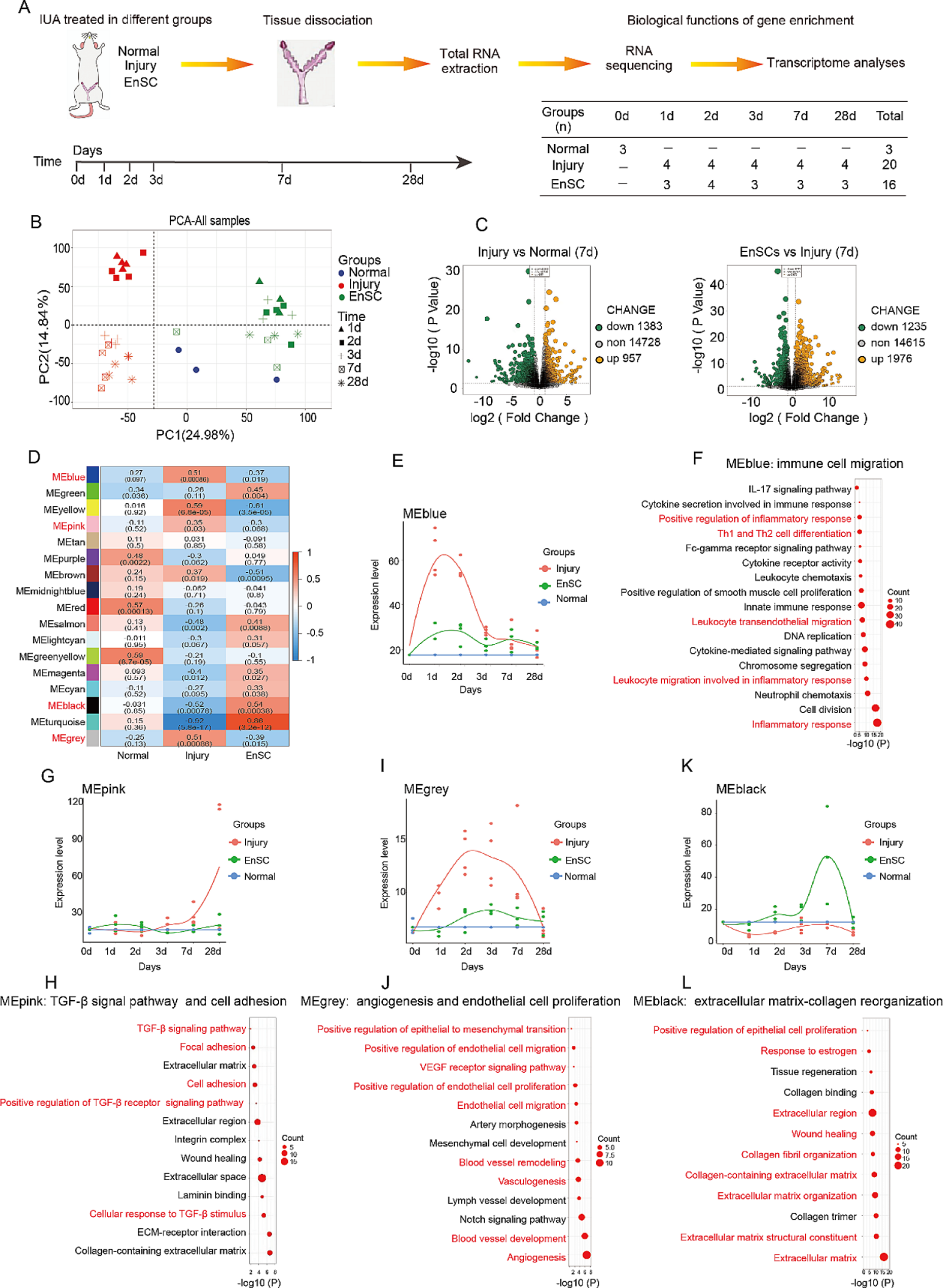
Transcriptome analysis of EnSCs transplantation in rat uteri with IUA. **A** A schema describing uteri samples from three groups for subsequent transcriptome analysis. **B** Principal component analysis (PCA) of uterine samples from various rat groups across different time points. **C** The volcano plot for the differentially expressed genes between the injury group vs. normal group (957 upregulated and 1383 downregulated genes) on the left panel, and between EnSC group and injury group (1976 upregulated and 1235 downregulated genes) on 7d post-injury on the right panel. **D** Heatmap of the correlation between the weighted gene coexpression network modules and the samples from three different groups. **E**, **G**, **I**, **K** The time-dependent expression profiles across all time points for the WGCNA change curves of MEBlue (**E**), MEpink (**G**), MEgrey (**I**), and MEblack (**K**) modules in the three different groups of samples. **F**, **H**, **J**, **L** Representative Gene Ontology (GO) functional terms in MEBlue (**F**), MEpink (**H**), MEgrey (**J**), and MEblack (**L**) modules. The GO functional terms were related to immune cell migration in the MEBlue module (F), TGF-β signal pathway and cell adhesion in the MEpink module (**H**), angiogenesis and epithelial cell proliferation in the MEgrey module (**J**) and extracellular matrix-collagen reorganization in the MEblack (**L**)

To decipher the potential mechanisms of recovery, rats in the injury group and the EnSCs group were euthanized on 1, 2, 3, 7 and 28 days, respectively. Uteri were collected to perform RNA-sequencing to identify potential genes and gene modules involved in the effective therapy (Fig. 5A). Two-dimensional principal component analysis (PCA) with normalized data matrix from all samples showed a clear separation of the different treatment samples (Fig. 5B). The result from principal component 1(PC1), representing 24.98% of variance, showed that EnSCs transplantation group was closer to normal endometrium, but distant from the control injury endometrium in gene expression profile of the endometrium (Fig. 5B). The result from principal component 2(PC2), accounting for 14.84% of the variance, showed that EnSCs-treated endometrium gradually approaches normal endometrium over time in gene expression profile (Fig. 5B). The PCA results revealed that EnSCs had obviously beneficial effects on restoring injured endometrium. Differentially expressed gene (DEG) analysis of 7 days samples revealed that the injury results in changes of 2,340 genes (957 upregulated genes and 1383 downregulated genes), compared to the normal group, while the EnSCs treatment induced 3,211 gene changes (1976 upregulated genes and 1235 downregulated genes) in comparison with the injury group (Fig. 5C). To uncover key genes involved in alleviation of fibrotic endometrium caused by EnSCs and promotion of endometrium regeneration, we identified 366 genes that were significantly upregulated post-injury but were dramatically inhibited after EnSCs treatment at 7 days (Fig. S4A) and 826 genes that were significantly downregulated post-injury but were dramatically upregulated after EnSCs treatment at 7 days (Fig. S4B). The upregulated genes in the injury endometrium were primarily involved in profibrotic extracellular matrix genes and Wnt, Hippo and MAPK signaling pathway genes (Fig. S4C). Interestingly, these differentially expressed genes included the transcription factor Jund, known to mediate TGF-β-induced fibroblast activation [29] (Fig. S4C), which suggests that EnSCs transplantation could potentially reverse the transition of fibroblasts to myofibroblasts. In contrast, upregulated genes in the EnSCs treatment endometrium were mainly associated with genes involved in the Estrogen pathway, fibroblast proliferation and cell migration (Fig. S4D).

To further confirm these results, we conducted weighted gene co-expression network analysis (WGCNA) based on the normal group, the EnSCs group and the control injury samples across all time points (Fig. S4E, F). WGCNA finally identified 17 modules after merging similar modules (Fig. 5D and S4G). Four modules exhibited obvious relationships with the therapeutic benefits caused by EnSCs transplantation (Fig. 5D). Three modules (MEBlue, MEpink, MEgrey) exhibited downregulated gene expression due to EnSCs transplantation, and MEblack module exhibited upregulated gene expression following EnSCs treatment (Fig. 5D). GO analysis showed that these genes in the MEBlue, MEpink and MEgrey module were related to immune cell migration and response, TGF-β signaling pathway, epithelial- mesenchymal transition, angiogenesis and endothelial cell proliferation, respectively (Fig. 5E-J). Notably, angiogenesis, which is necessary for tissue repair, appeared a slight upregulation in the EnSCs group, whereas in the injury group, angiogenesis was significantly upregulation of in early stage and subsequently downregulated in the late stage. Although the reason for significantly upregulated of angiogenesis in the injury endometrium is unclear, we speculate that one possible explanation is that injury-induced inflammation may induce angiogenesis in a maner similar to injury caused by menstruation, whereas EnSCs treatment may inhibit inflammation, resulting in only slight angiogenesis. MEblack genes were associated with extracellular matrix and collagen reorganization, responding to estrogen and epithelial cell proliferation (Fig. 5K, L). These results suggested that EnSCs grafts brought immunosuppressive effects during the early stages of injury (Fig. 5E, F), while in the later phases of injury, they mainly regulated extracellular matrix remodeling to resist fibrosis (Fig. 5K, L). Furthermore, EnSCs transplantation inhibited the pro-fibrotic transforming growth factor beta (TGFβ) pathway and activated estrogen signaling in vivo, which could play a significant role in endometrial regeneration (Fig. 5G, H and S4H). Next, we also used differentially expressed genes of injury versus normal samples and EnSCs versus injury samples to conduct functional enrichments with Gene Set Enrichment Analysis (GSEA), and analyzed their representative pathways based on their NES (Normalized Enrichment Score) results, respectively (Fig. S4H-J). The NES results indicated the EnSC grafts regulated the estrogen signaling pathway, cell adhesion molecules, adherens junction and immunoregulation ability (Fig. S4H-J).

**Fig. 6:**
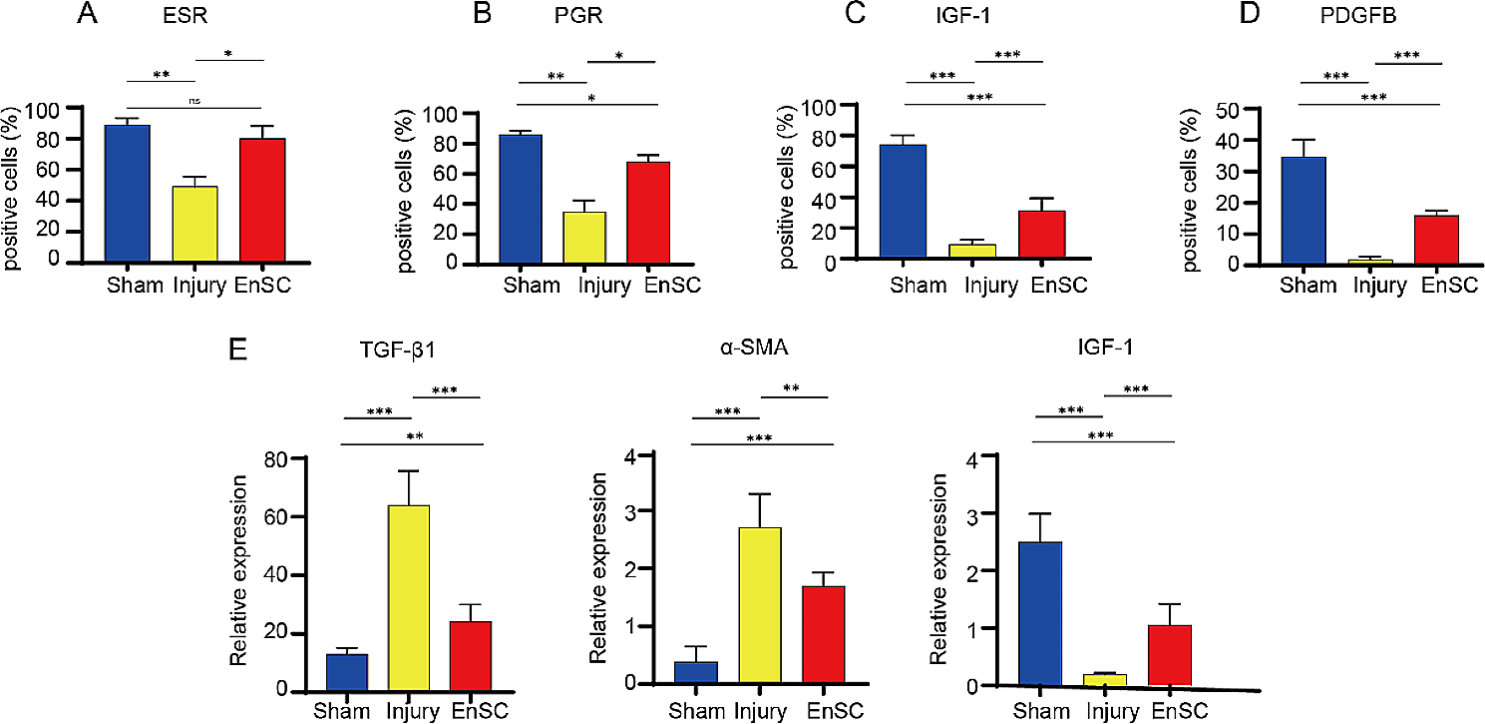
EnSCs transplantation increased regenerative molecules and decreased key pro-fibrotic maker. **A**-**D**. Quantification of ESR-positive cells (**A**), PGR-positive cells (**B**), IGF-1-positive cells (**C**), and PDGFB-positive cells (**D**) in the endometrium of different groups using immunostaining on the 7th day post-injury. Data are represented as mean ± SD (*n* = 8). **E** Quantification of *TGF-β1*, *α-SMA* and *IGF-1* expression in the uterus of different groups on the 7th day post-injury using quantitative RT-PCR. Data are represented as means ± SD (*n* = 8). One-way ANOVA. *p < 0.05, **p < 0.01, ***p < 0.001, ns, no significance

To further confirm transcriptome results, we next performed uterus tissue immunostaining. Given that ESR and PGR play critical roles in regulating endometrium regeneration, we detected expression of ESR and PGR by immunostaining at 7 days post-EnSCs transplantation. In contrast to the control IUA rats, a marked increase of ESR and PGR proteins was detected in the EnSCs-grafted IUA rats (Fig. 6A, B and S5A, B). Notably, in the EnSCs-transplanted group, the endometrial epithelium regeneration factor IGF1, originating from stromal cells [30], and the tissue remodeling growth factor PDGFB were significantly upregulated (Fig. 6C, D, and S5C, D). Besides, the marker CD31, a well-defined marker of angiogenesis, is also upregulated (Fig. S5E). These results implied that repair of endometrium might be partially dependent on paracrine growth factors released by EnSCs. Real-time PCR results also showed a downregulation of pro-fibrotic factors TGFβ1 and α-SMA and an increase in regenerative factor IGF1 following EnSCs transplantation (Fig. 6E). In conclusion, the restoration of the endometrium after EnSCs transplantation is based on their anti-inflammatory effects, anti-fibrotic properties and regenerative factor secretions that stimulate epithelialization.

## Discussion

The endometrium consists of a functional layer and a basal layer. The functional layer is a highly organized mucosal layer that undergoes monthly cyclical changes and is shed during menstruation [31]. The basal layer is hypothesized to be the residence of both epithelial and stromal adult stem cells, which play a pivotal role in the regeneration of the endometrium [32, 33]. Increased artificial abortions and other intrauterine surgeries have been one of the main drivers of IUA cases around the world [34]. In this study, we successfully isolated EnSCs from the endometrium of human donors. These EnSCs could be largely expanded in vitro and maintained MSC characteristics, immunoregulatory functions and sex hormone responsiveness. Importantly, xenogeneic transplantation of EnSCs successfully repaired the injured endometrium and significantly improved the pregnancy rate in IUA rats.

The damage to the endometrium disrupts cell-to-cell communication, thereby disturbing the normal process of endometrial regeneration and leading to dysfunctional endometrium. It is widely accepted that persistent inflammation and disrupted cellular communication due to an imbalance in the estrogen-progesterone axis are two primary factors involved in the formation of IUA [35, 36]. Therefore, the current treatment strategy for intrauterine adhesions primarily relies on the anti-inflammatory effects of MSCs and the administration of estrogen to rebuild the endometrial ecological environment, thereby regenerating the endometrium [37–39]. In addition, most of the seed cells used for treating IUA primarily rely on paracrine effects to downregulate tissue fibrosis-related TGF-β signaling [35, 40]. Based on the above treatment strategies, it is believed that the ideal therapeutic seed cells for IUA should be capable of regulating inflammation, responding to hormones and inhibiting fibrosis-related TGF-β signaling. EnSCs have prominent advantages in regenerative medicine. Similar to MSCs from other sources such as umbilical cord, adipose tissue, and bone marrow, EnSCs possess both anti-inflammatory properties and trilineage differentiation potential [17–19]. Importantly, EnSCs possess unique characteristics in responding to hormones from the uterine endometrial niche, which may be a key factor for endometrial regeneration [41]. Bioinformatics analysis revealed that EnSCs not only downregulated inflammation responses and the TGF-β signaling pathway but also activated genes related to the estrogen signaling pathway after transplantation. Estrogen is often used alone or in combination with various types of seed cells for IUA treatment, as it is believed to be crucial in activating basal layer stem cells. These findings emphasize the advantages of EnSCs as seed cells in treating endometrial fibrosis.

Recently, understanding the immunomodulatory plasticity of stromal cells has been of great importance for their application in tissue regeneration. In our study, the IFN-γ-induced transcriptome of EnSCs showed that the spectrum of immunomodulatory molecules mainly cluster in antigen presentation, chemokine signaling pathway and interferon signaling pathways. Other immunomodulatory factors, such as CCL2, CCL5, CCL7, CXCL9, CXCL10 and CXCL11 chemokines that are involved in the recruitment and regulation of immune cell function, were detected with enhanced expression at the mRNA level. These findings suggest that EnSCs can act as antigen-presenting cells in response to inflammatory signals. Furthermore, they upregulated surface markers that regulate immune cell function, such as costimulatory and immunosuppressive molecules. They also secreted various factors, including interleukins and chemokines, to mobilize immune cells in the surrounding microenvironment. Traditionally, it is believed that the expression levels of PD-L1/PD-L2 and IDO1 usually predict the therapeutic value of MSCs in ameliorating human disease [42]. In this study, we observed that EnSCs exhibited great similarity to MSCs in the response properties to IFN-γ, but EnSCs possess the tissue-specific immune properties. Similarly, IFN-γ treatment was able to upregulate HLA-class I and II molecules on the cell surface of both MSC [43] and EnSCs at the RNA level. Unlike MSCs from other tissue sources, endometrial tissues are powerful regenerative tissues regulated by estrogen, which is well known for its immunomodulatory properties [44, 45]. Therefore, investigating the immunomodulatory properties of EnSCs may need to be explored in an estrogenic context in the future.

Although both bulk RNA-seq transcriptome of EnSCs primed by IFN-γ in vitro and EnSCs transplantation in vivo suggest that EnSCs have an immunomodulatory role, it is not well understood how immune cell function is regulated by EnSCs. MSCs have been shown to participate in both innate and adaptive immunity by interacting with immune cells, including T cells, B cells, natural killer (NK) cells, macrophages, monocytes, dendritic cells (DCs) and neutrophils, via cell-to-cell contact and paracrine activity [46]. Thus, in order to reveal the function of EnSCs on immune cells, future studies should include co-culture experiments of EnSCs with immune cells in vitro. The secretome of EnSCs is a diverse repertoire of multifaceted cytokines, growth factors, and chemokines, which combine to modulate the function of immune cells. For example, IL-6 has been shown to protect neutrophils from apoptosis [47]. IL15 is a key cytokine driving uNK cell differentiation, the latter successively enhancing angiogenic capacity [48]. Previous studies show that the three CXCL9-, 10- and 11-CXCR3 axes predominantly driven by IFN-γ, regulate immune cell migration, differentiation and activation, especially shifting naive T cells to T helper 1 (Th1) cells [49]. In addition, the CCL2- and 7-CCR2 and CCL5-CCR5 chemokine axes play a crucial role in recruiting monocytes/macrophages to injury sites [50], which is key to tissue regeneration. These results indicate that although pro-inflammatory factors were induced by IFN-γ treatment, they may facilitate the endometrium regeneration. Future research should explore the key factors secreted by EnSCs to participate in regeneration. So far, many cellular strategies for IUA treatment have mainly focused on naked MSC cells. After fully revealing the immune-regulatory function of EnSCs, future cellular therapies may require engineered EnSCs to enhance their immune function for better antifibrotic effects.

Recently, single cell sequencing of the endometrium revealed that stromal cells are heterogeneous [51, 52]. Several studies have reported a subpopulation of stromal cells in the endometrium with immunoregulatory properties. In the mouse endometrium, GFP-positive mesenchymal cells from *Pdgfrb*-BAC-eGFP transgenic mice were annotated into two populations of perivascular cells (KCNJ8^+^ pericytes and CNN1^+^ vascular smooth muscle cells) and three populations of fibroblasts (NGFR^+^ type I, CXCL14 ^+^ type II and CLEC3B^+^ type III) by single-cell sequencing [52]. These three types of fibroblasts were characterized for immune response, wound healing, and extracellular matrix remodeling, respectively [52]. Single-cell sequencing of full-thickness normal human endometrium uterine from proliferative and secretory phases revealed four populations of stromal cells, secretory stroma (SCGB1D2), SFRP4^+^ stroma (SFRP4), DCN^+^ stroma (DCN) and inflammatory stroma (IL6) [30]. In our reported human endometrial assembloids with a luminal epithelium, EnSCs were identified into six endometrial stromal cells populations, of which the EnSC subpopulation 2 (EsS2) is positive of CXCL8, CXCL1 and IL6 genes and exhibits immunoregulatory properties [53]. These studies suggest that immunoregulation of stromal cells may be exerted by some specific stromal cell subpopulations, and different stromal cell subpopulations are likely to play different roles in endometrial regeneration. Previous studies have reported that the highly enriched SFRP4^+^ stromal cell subpopulation in the proliferative phase of the endometrium, which exhibits high expression of the IGF1 signaling pathway, can promote the proliferation of endometrial epithelial cells [30]. Therefore, it may be important to develop the appropriate unified markers for sorting out endometrial subtypes of stromal cells and reveal their therapeutic mechanisms for treating IUA in the future.

Although this study has evaluated the efficacy of EnSCs in treating uterine adhesions and has preliminarily demonstrated that the therapeutic efficacy of EnSCs is mainly dependent on their anti-inflammatory, antifibrotic, and pro-regenerative effects, the specific mechanism underlying the effectiveness of EnSCs has not yet been revealed. The development of serum-free culture methods is needed for the future application of EnSCs in the clinic.

Overall, EnSCs transplantation has promoted the recovery of the endometrium affected by intrauterine adhesions, as well as the treatment of endometrial fibrosis and the enhancement of fertility. This therapeutic effect may be closely related to the immunomodulatory and anti-fibrotic effects of EnSCs. Our study could offer a potential treatment avenue for injury induced IUA.

## Conclusions

In summary, this study revealed that EnSCs could be used as donor cells to treat IUA. In vitro, EnSCs possess the ability to undergo trilineage differentiation (adipogenic, chondrogenic, and osteogenic differentiation), remarkable immunomodulatory capabilities after IFN-γ stimulation and hormone-responsiveness. In vivo, EnSCs transplantation can promote the structural and functional recovery of the endometrium and improve the pregnancy rate. Mechanistically, the anti-inflammatory, anti-fibrotic, and regenerative factor-secretion properties of EnSCs can promote endometrial regeneration.

### Electronic supplementary material

Below is the link to the electronic supplementary material.

Supplementary Material 1

## Data Availability

The data reported in this paper have been deposited in the OMIX, China National Center for Bioinformation/Beijing Institute of Genomics, Chinese Academy of Sciences (https://ngdc.cncb.ac.cn/omix: accession no. OMIX006591 and OMIX005741).

## Abbreviations

IUA: Intrauterine adhesion
EnSCs: Endometrial stromal cells
MSCs: Mesenchymal stem cells
MEnSCs: Menstrual blood-derived stromal cells
NK: Natural killer cells
DCs: Dendritic cells
Th1: T helper 1
TGFβ: Transforming growth factor beta
GSEA: Gene Set Enrichment Analysis
NES: Normalized Enrichment Score
