# Supplementary material for: Restoration of functional endometrium in an intrauterine adhesion rat model with endometrial stromal cells transplantation
Source: Stem Cell Res Ther. 2024 Jun 21;15:181. doi: 10.1186/s13287-024-03788-z (PMC11191336; doi:10.1186/s13287-024-03788-z)
Supplement: Supplementary file 1 — Supplementary Material 1 [file 13287_2024_3788_MOESM1_ESM.docx]

**Restoration of functional endometrium in an intrauterine adhesion rat model with endometrial stromal cells transplantation**


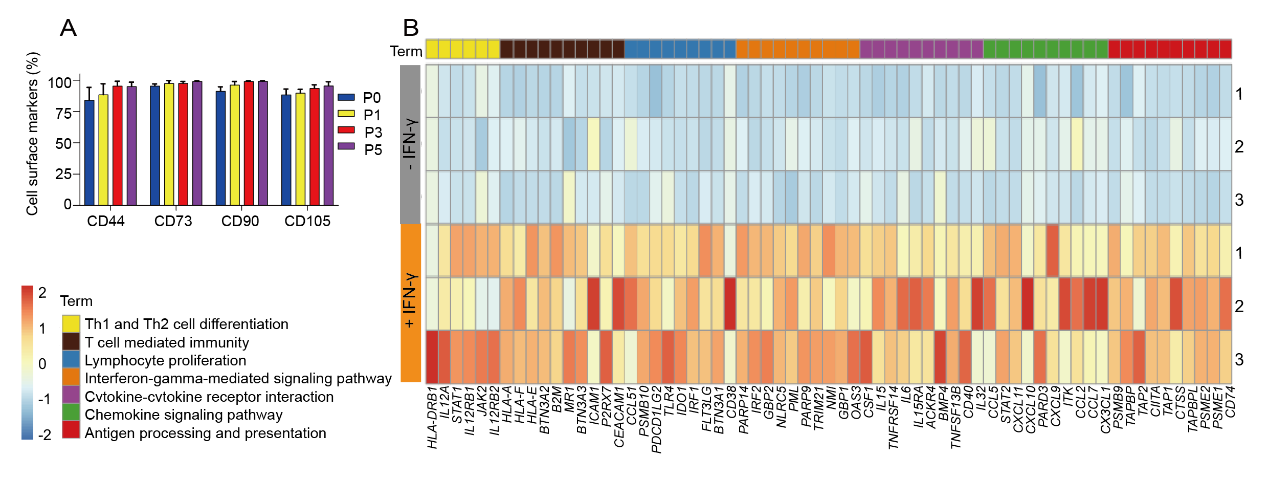


**Supplementary Figure 1** The biological properties of EnSCs, relative to Figure 1. **A** Quantification of mesenchymal stromal cell markers in EnSCs over passaging by flow cytometry. n=5. **B** The heatmap of the key genes in IFN-γ-treated EnSCs, compared to in the non-treated control EnSCs (n=3).


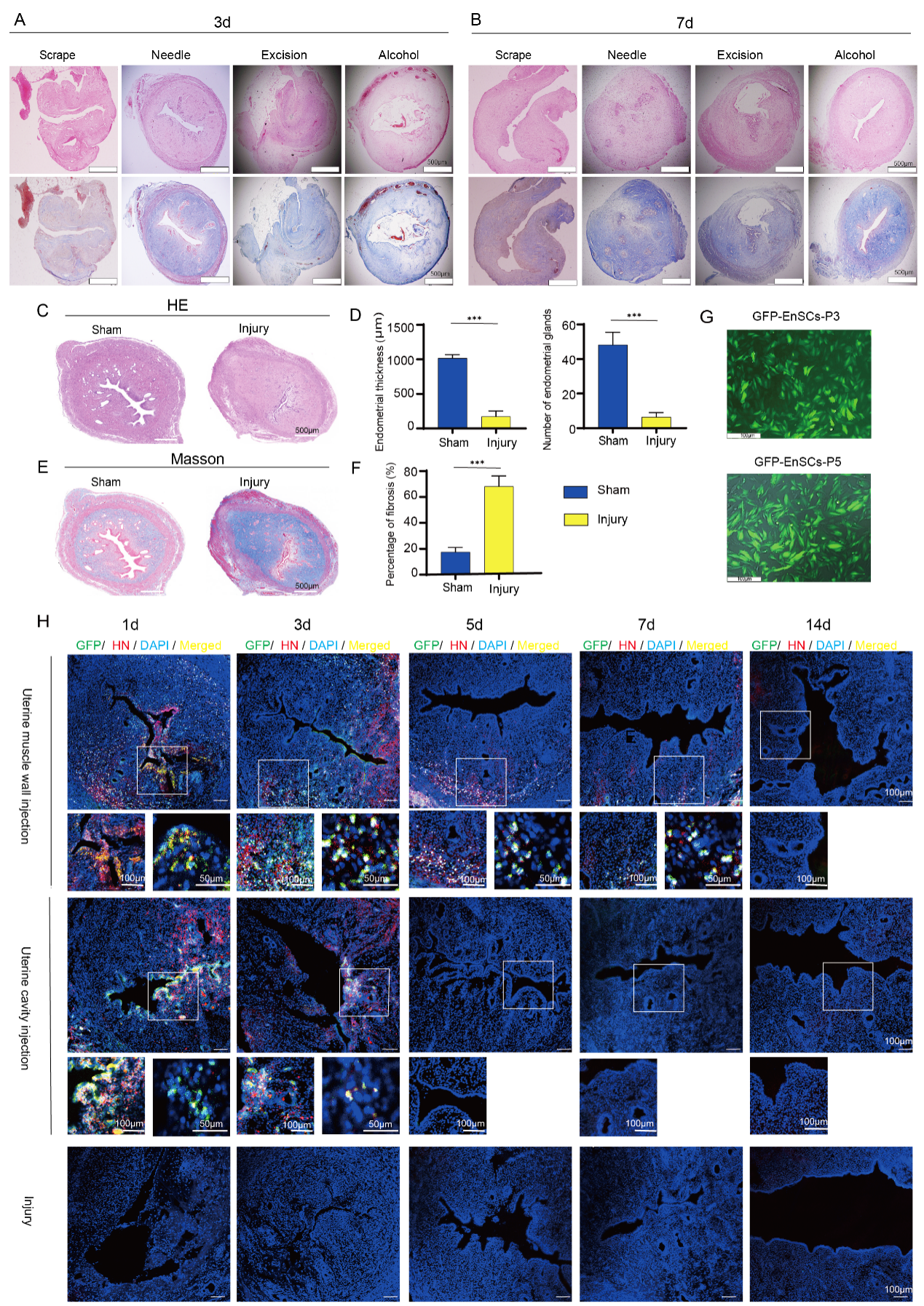


**Supplementary Figure 2** Establishment the IUA model and comparing of the GFP-EnSC transplantation injections to determine the most suitable cell delivery method. **A, B** Evaluation of an effective and stable IUA model among four modeling methods (Needle, Scrape, Excision, Alcohol) using HE and Masson staining at 3 days (A) and 7 days (B), n=6. Scale bars: 500µm. **C, E** Figure (C) illustrates the H&E staining and (E) represents the Masson’s trichrome staining of uteri comparing the sham-operated group with the injury group on the 7th day after needle scraping. Scale bars: 500µm. **D, F** Statistical analysis comparing endometrial thickness, endometrial glands (D), and percentage of fibrosis (F) between the sham-operated group and the injury group on the 7th day after uterine scratching, Data are represented as means ± SD (n = 6). Unpaired Student’s t test. ***p < 0.001. **G** GFP-labeled EnSCs (P3 and P5) observed under fluorescence microscopy after 48 hours. Nearly 90% of the cells exhibited transfection, displaying intense green fluorescence. Scale bars: 100µm. **H** GFP (green), human nuclear antibody (HN, red), DAPI (blue), and the merge (yellow) were detected using confocal microscopy in the uterine muscle wall injection group, uterine cavity injection group and the injury group at 1, 3-, 5-, 7-, and 14-days post-injury. Scale bars: 100µm and 50µm.


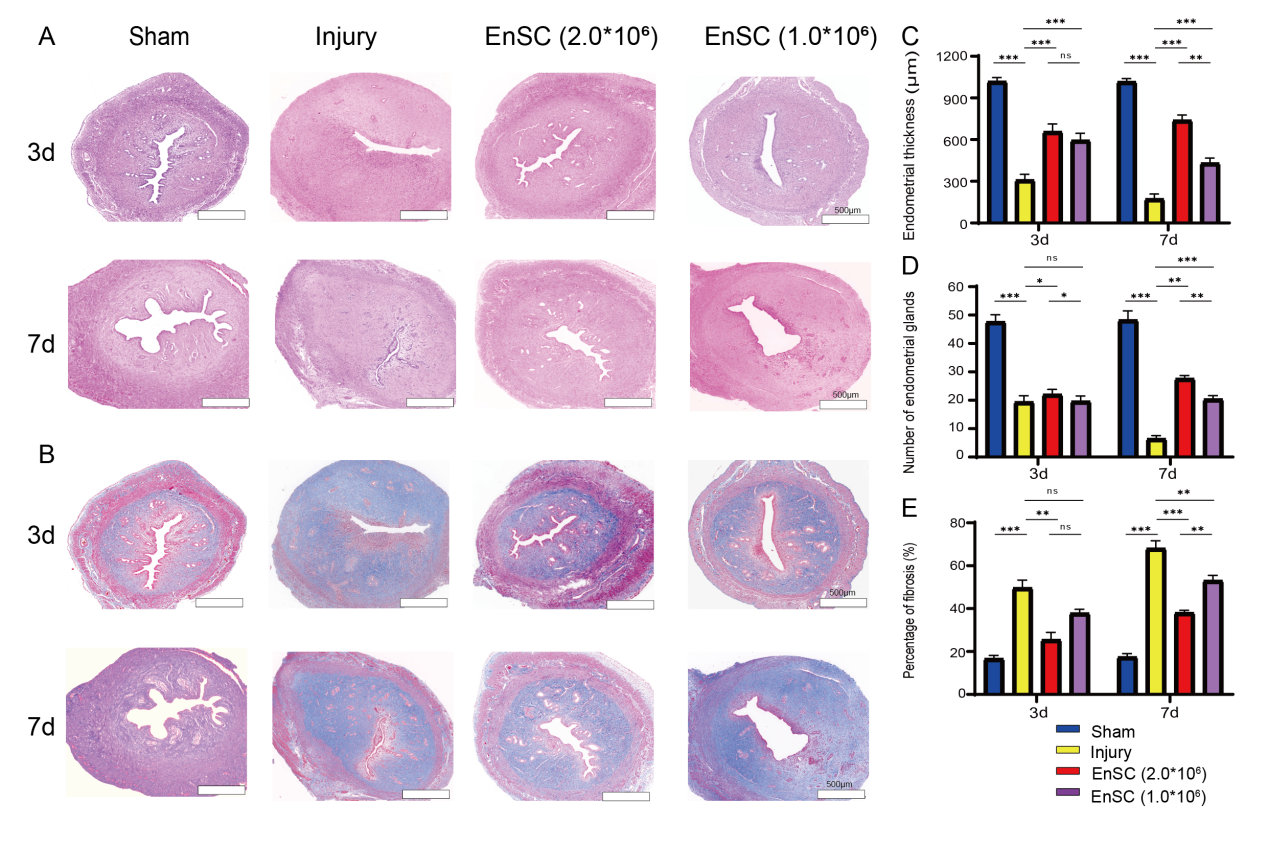


**Supplementary Figure 3** Comparison of EnSC transplantation efficacy at different cell doses. **A, B** H&E staining (A) and Masson’s trichrome staining (B) of uteri in the sham, the injury control group and EnSC-treatment groups rats transplantaed with different doses (2.0*106 or1.0*106 cells per rat uterus) on 3-,7-d post-injury, respectively. Scale bars: 500µm. **C D E** Quantification of endometrial thickness (C), endometrial gland numbers (D) and fibrosis degree (E) in rats from the injury group and the EnSC treatment group at 3-, 7-days post-injury. Data are means ± SD (n = 6). One-way ANOVA. *p < 0.05, **p < 0.01, ***p < 0.001, ns,no significance.


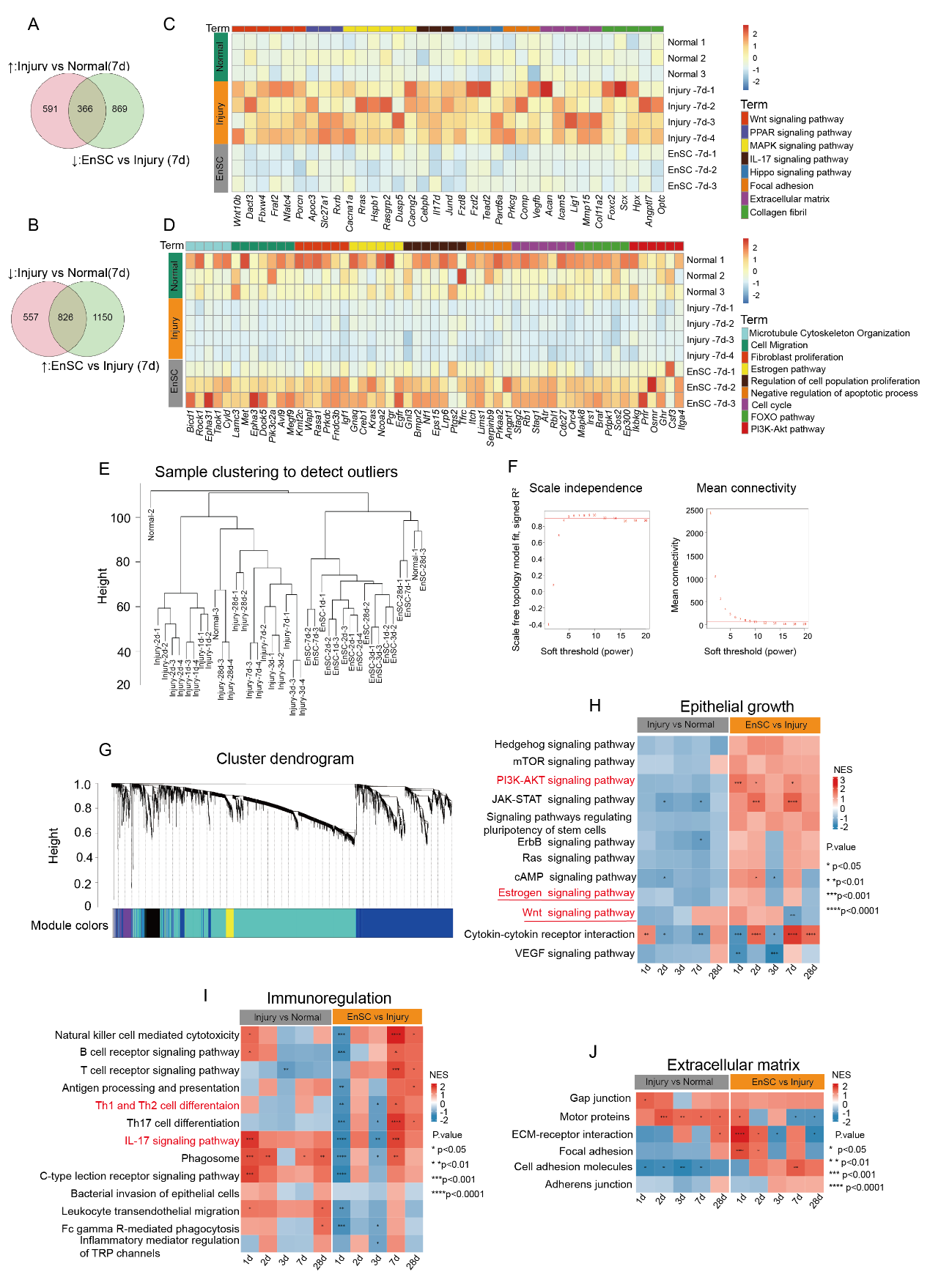


**Supplementary Figure 4** Weighted gene coexpression network construction (WGCNA) and Gene Set Enrichment Analysis (GSEA) of the rat uteri. **A, B** Venn diagram showed the differential transcribed genes between Injury vs Normal （red circle）and EnSC vs Injury (green circle) 7 days post-injury. Arrow up marked upregulated and arrow down marked downregulated genes after post-injury or EnSC treatment, and got 366 (A) and 826 (B) intersection genes respectively. **C, D** The heatmap of the key genes from the 366 (C) and 826 (D）intersection genes 7 days post-surgery between Injury, Normal and EnSC. **E** The sample clustering dendrogram of all samples across all time points in weighted gene co-expression network analysis (WGCNA). **F** Soft thresholding power was obtained by analyzing the scale-free fit index and mean connectivity for different soft threshold powers. **G** The module clustering tree diagram showed the modules based clustering, which remained 17 modules after merging similar modules. **H, I, J** The enrichment score (NES) heat map of GSEA of the KEGG functional enrichment pathway selected from three aspects, namely epithelial proliferation (H), immune regulation (I), and extracellular matrix(J). The x-axis is the comparison of different groups at different time points (1d, 2d, 3d, 7d, and 28d). The y-axis is the KEGG pathway. Each cell represents the NES of pathway in the corresponding group.


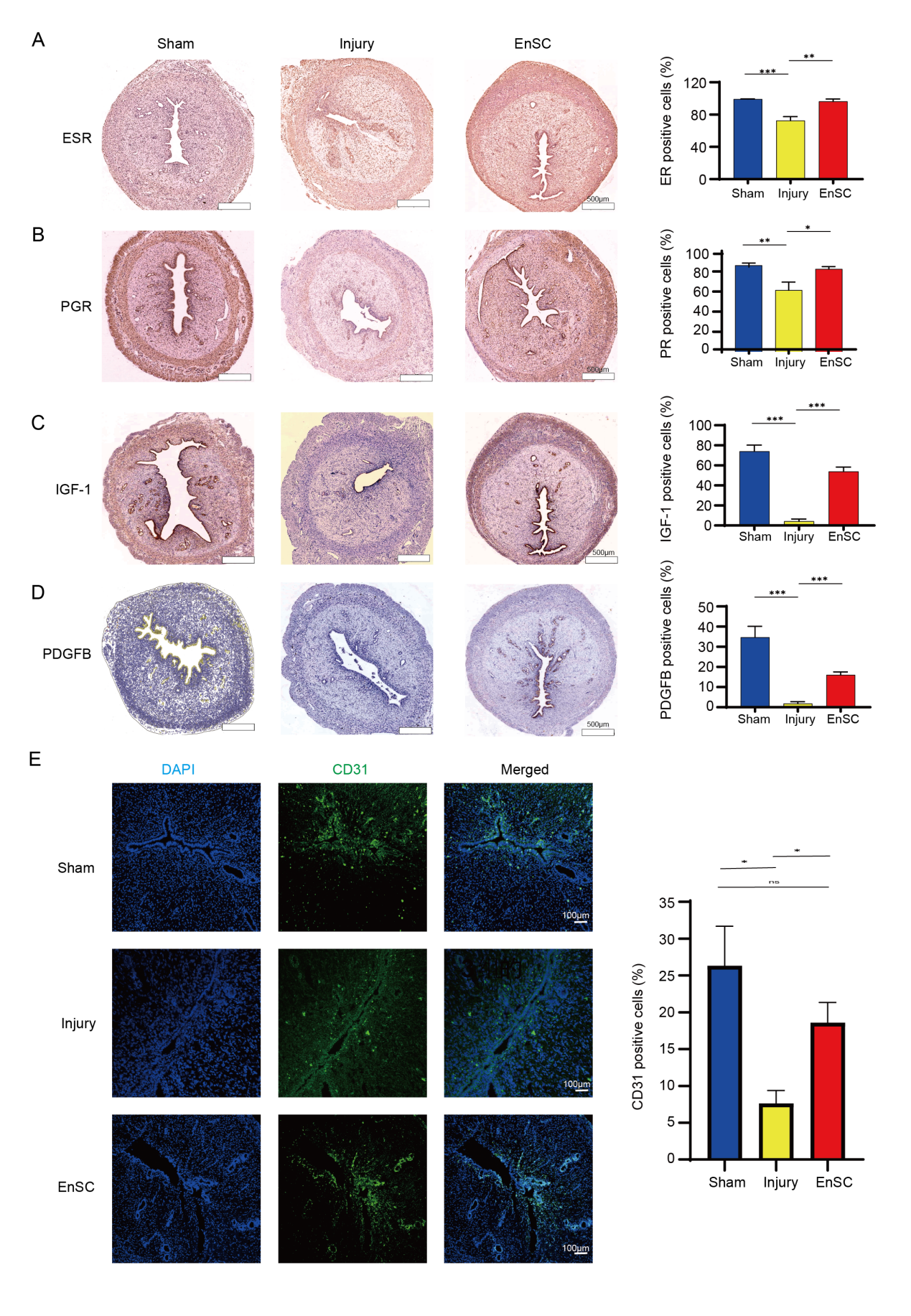


**Supplementary Figure 5** EnSCs transplantation increased the regenerative molecules. **A, B, C, D** Immunohistochemical staining and quantification of ESR (A), PGR (B), IGF-1(C), PDGFB(D) was performed in the different groups at 7 days post-injury (Scale bar: 500 µm). Data are represented as means ± SD (n = 6). One-way ANOVA. *p < 0.05, **p < 0.01, ***p < 0.001. **E** Representative immunostaining images and quantification of CD31 in uteri from different groups at 7 days post-injury (Scale bars: 100µm). Data are represented as means ± SD (n = 6). One-way ANOVA. *p < 0.05, ns, no significance.

Supplementary table 1. The information of 5 donor patients for isolation of EnSCs.

| Donor | Age  (years) | BMI | Menstrual  regularity | Menstrual phase | Gynecological  disorder | Estradiol concentration  (E2) pmol/l | Hysteroscopy of endometrium /Histological diagnosis |
| --- | --- | --- | --- | --- | --- | --- | --- |
| 1 | 31 | 23.8 | Regular | Proliferative | Uterine myoma | 113.5 | Normal |
| 2 | 28 | 25.6 | Regular | Proliferative | Infertility  (fallopian tube lockage) | 128.7 | Normal |
| 3 | 25 | 19.8 | Regular | Proliferative | Uterine myoma | 98.7 | Normal |
| 4 | 29 | 22.7 | Regular | Proliferative | Infertility  (fallopian tube lockage) | 105.5 | Normal |
| 5 | 35 | 24.3 | Regular | Proliferative | Ovarian teratomas | 84.6 | Normal |

BMI, body mass index.

Supplementary table 2. Primers of each target gene used in Quantitative RT-PCR analyses

| Gene |  | Sequence(5’-3’) | Nucleotide  base |
| --- | --- | --- | --- |
| IGFBP1 | Forward primer | TCCTTTGGGACGCCATCAGTAC | 22 |
|  | Reverse primer | GATGTCTCCTGTGCCTTGGCTA | 22 |
| PRL | Forward primer | AAGCTGTAGAGATTGAGGAGCAAAC | 25 |
|  | Reverse primer | TCA GGA TGA ACC TGG CTG ACT A | 22 |
| GAPDH  (human) | Forward primer | CAGGAGGCATTGCTGATGAT | 20 |
|  | Reverse primer | GAAGGCTGGGGCTCATTT | 18 |
| IGF1 | Forward primer | AGATGTACTGTGCTCCGCTG | 20 |
|  | Reverse primer | TTCTTGTGTGTCGATAGGGGC | 21 |
| TGFB-1 | Forward primer | CACTCCCGTGGCTTCTAGTG | 20 |
|  | Reverse primer | GGACTGGCGAGCCTTAGTTT | 20 |
| α-SMA | Forward primer | AGACCCTCTTCCAGCCATCT | 20 |
|  | Reverse primer | CCCCGAGAGGACGTTGTTAG | 20 |
| GAPDH  (rat) | Forward primer | GATGCTGGTGCTGAGTATGRCG | 22 |
|  | Reverse primer | GTGGTGCAGGATGCATTGCTCTGA | 24 |
